# SARS-CoV-2 Prevalence in Laparoscopic Surgery Filters. Analysis in Patients with Negative Oropharyngeal RT-qPCR in a Pandemic Context: A Cross-Sectional Study ^†^

**DOI:** 10.3390/jpm11111052

**Published:** 2021-10-20

**Authors:** Antoni Llueca, Manuela Barneo-Muñoz, Javier Escrig, Rosa de Llanos, on Behalf of COVID-Lap Working Group

**Affiliations:** 1 Multidisciplinary Unit of Abdominal Pelvic Oncology Surgery (MUAPOS), University General Hospital of Castellon, 12071 Castellón, Spain; 2 Department of Medicine, University Jaume I (UJI), 12071 Castellon, Spain; barneo@uji.es (M.B.-M.); escrigv@uji.es (J.E.); dellanos@uji.es (R.d.L.)

**Keywords:** SARS-CoV-2, laparoscopy smoke evacuation, laparoscopy filters, COVID-19, laparoscopic surgery

## Abstract

Objective: Surgical societies of different specialties have lately demonstrated a growing concern regarding the potential risk of SARS-CoV-2 transmission during surgery, mainly via aerosols carrying SARS-CoV-2 particles during laparoscopy smoke evacuation. Since there is not sufficient scientific evidence to rule out this hypothesis, our study aimed to evaluate the prevalence of the appearance of SARS-CoV-2 genetic material in the in-filter membrane of the smoke filter systems, used in laparoscopic surgery, in a tertiary referral hospital during the peak phases of the pandemic. Methods: During the highest incidence of the pandemic outbreak, 180 laparoscopic smoke evacuation systems were collected from laparoscopies performed between April 2020 and May 2021 in University General Hospital of Castellón. As part of the safety protocol established as a result of the pandemic, an oropharyngeal reverse-transcription polymerase chain reaction (RT-PCR) was performed before surgery. We performed RT-qPCR tests for the detection and quantification of SARS-CoV-2 genetic material in the in-filter membranes extracted from the smoke evacuation systems. Results: We found two RT-qPCR positive in-filters from a sample of 128 patients with SARS-CoV-2-negative results in their oropharyngeal RT-qPCR, i.e., 1.6% (95% CI: 0.5–5.5%). From this estimation, the predictive posterior probabilities of finding *n* cases of negative oropharyngeal COVID-19 patients with positive filters increases with the increasing number of surgeries performed. Conclusions: This cross-sectional study provides evidence suggesting that airborne transmission of SARS-CoV-2 particles from smoke evacuation of aerosols carrying viral particles during laparoscopy should not be ruled out.

## 1. Introduction

The COVID-19 pandemic caused by SARS-CoV-2 has put the health systems of all countries worldwide to the test, as well as the surveillance and diagnosis systems of the disease, in addition to a strong economic and social crisis, the consequences of which are still to come. Faced with situations such as the current one, numerous doubts and uncertainties arise in health systems around the world, in relation to the safety measures to be taken during routine hospital activities, such as surgical interventions, including those carried out laparoscopically.

Under normal circumstances, laparoscopy is a safe surgical procedure that creates a physical barrier between the surgeon and the potential source of infection (the patient), thus avoiding occupational exposure and cross-contamination. However, in a health crisis such as the COVID-19 pandemic, caused by a respiratory infection, the risk of transmission of SARS-CoV-2 during laparoscopy, although theoretical, must be seriously considered. The main route of transmission of SARS-CoV-2 is airborne through respiratory droplets (aerosols) and direct contact, without ruling out the faecal–oral route. For this reason, some studies suggest the possibility of viral contamination taking place during laparoscopy, through the possible release of the virus in the form of aerosols and fluids that are generated during surgical procedures [1]; therefore, it is worth considering that there is a risk of air contamination in the operating room given the emptying of the pneumoperitoneum at the end of laparoscopic surgery. Before the current pandemic, laparoscopic gas evacuation used to be performed freely without the interposition of safety filters. The lack of precise data on the viral load in the different compartments and body fluids forced healthcare personnel to work in a situation of uncertainty and insecurity.

In fact, the mechanisms for the excretion of SARS-CoV-2 in the surgical context and its consequences on infections are largely unknown. Until now, there has been no scientific evidence in this regard, and there are only recommendations that the clinical–scientific community has raised on the basis of previous experiences with other highly transmissible viruses through surgery, such as Ebola [2], as well as evidence related to the presence of hepatitis B virus in the pneumoperitoneum during laparoscopy [3]. Some authors have recently reported cases where SARS-CoV-2 has been detected in peritoneal fluids, blood, and cerebrospinal fluid [4,5,6,7,8].

Consequently, different specific surgical societies around the world [8,9,10] have acted quickly to establish general recommendations, especially based on expert opinions, for performing surgical interventions, such as the use of personal protective equipment and training health personnel. In the specific case of laparoscopic interventions, the protection measures of the airway and mucous membranes are extreme, and the use of filters for the evacuation of CO_2_ after surgery has also been recommended.

The objective of this cross-sectional study is to evaluate the prevalence of the appearance of SARS-CoV-2 genetic material in the in-filter membrane of the smoke filter systems, used in laparoscopic surgery, in a tertiary referral hospital during the peak phases of the pandemic.

## 2. Materials and Methods

### 2.1. Materials

The laparoscopic smoke evacuation systems used in each patient surgery intervention were collected at the end of the laparoscopies performed between April 2020 and May 2021 in General University Hospital of Castellón, in a setting of a high population risk of transmission (cumulative incidence in 7 days of 75–125/100,000) or extreme risk (cumulative incidence in 7 days >125/100,000). Smoke evacuation systems were properly identified and stored at −80 °C, following standard operating procedures in the General University Hospital of Castellón Unit of the IBSP-CV Biobank (PT17/0015/0017) integrated in the Spanish National Biobanks Network and in the Valencian Biobanking Network.

Due to conditioning factors of the hospital safety protocol for surgery, the majority of asymptomatic cases with negative oropharyngeal reverse-transcription polymerase chain reaction (RT-PCR) were intervened urgently or on a scheduled basis, as well as a minority of symptomatic positive oropharyngeal RT-qPCR cases whose intervention could not be postponed.

### 2.2. In-Filter Membrane Sampling and RNA Extraction

There are three main types of commercially available closed filtration systems collected from the laparoscopic procedures. During processing, smoke evacuation systems were first dismantled to allow in-filter membranes to be extracted. In-filter membranes were placed in a 50 mL tube and spiked with 10^2^ infective units of mengovirus vMC0 (CECT 100000) (MgV), a nonenveloped member of the Picornaviridae, used as an external recovery control (or also known as a process control) to understand the amount of virus lost during sample processing, as well as to evaluate the efficiency of RNA extraction, following a protocol similar to ISO 15216–2:2017 used in food products. This procedure has been previously validated for nonenveloped viruses [11,12]. In addition, 1 mL of lysis buffer (Nucleospin RNA virus Kit (Macherey-Nagel)) was added to the in-filter membranes followed by centrifugation for 10 min at 8500 rpm. From the initial 1 mL of lysis buffer, approximately 600–400 μL of solution was collected, which was processed for viral RNA extraction using the Nucleospin RNA virus Kit (Macherey-Nagel) following the recommended protocols, resulting in a final eluate of 70 μL.

Smoke filtration system manipulation, in-filter membrane collection, and RNA extraction were carried out in a Biosafety Level II cabinet.

### 2.3. SARS-CoV-2 RT-qPCR Detection

The presence of SARS-CoV-2 was determined by specific amplification and FAM-probe detection of N1 and N2 regions of the nucleocapsid gene, validated by the US Centre for Disease Control and Prevention [13] (2019-nCoV RUO Kit, Integrated DNA Technologies, Coralville, IA, USA), and the envelope gene E, validated by the Charité–Universitätsmedizin Berlin Institute of Virology from German Centre for Infection Research (Berlin, Germany) [14] and accepted by the World Health Organisation (E Assay First Line Screening; Integrated DNA Technologies, Geneva, Switzerland). The presence of human genetic material was determined by specific amplification and FAM-probe detection of Ribonuclease P protein subunit p30 (*RPP30*), a single-copy human gene (2019-nCoV RUO Kit; Integrated DNA Technologies). Mengovirus determination was also performed using specific primers and FAM-probe described in ISO 15216-1:2017. Reverse-transcription polymerase chain reaction (RT-PCR) was performed using the One Step PrimeScript^TM^ RT-PCR Kit (Perfect Real Time, Takara, Shiga, Japan) following the manufacturer’s instructions and carried out in a StepOnePlus Real-Time PCR System (Applied Biosystems, Foster City, CA, USA). Briefly, a first step of retrotranscription at 45 °C for 10 s, followed by 40 cycles of PCR amplification and fluorescence recording (consisting of a denaturing step at 95 °C for 5 s, and annealing/elongation at 55 °C for 34 s), was set up (CDC-006-00019, Revision: 06). SARS-CoV-2, *RPP30*, and MgV were determined for each in-filter membrane. In addition, negative and positive samples were also included in the assay to verify the specificity of amplifications. Nuclease-free water used in the RNA extraction protocol was used as a negative control (non-template control) with no fluorescence readings. Control plasmids containing the complete nucleocapsid and envelope genes (nCoV-N control and nCoV-E controls: severe acute respiratory syndrome coronavirus 2 isolate Wuhan-Hu-1, complete genome, GenBank: NC_045512.2; IDT)) were the positive samples. Standard curves for MgV and SARS-CoV-2 quantifications were performed using the genomic RNA of MgV and the 2019-nCoV_N_Positive Control for gen targets N1 and N2, and 2019-nCoV_E_Positive Control for gen target E, in triplicate and with serial 10-fold dilutions.

### 2.4. Statistical Analysis

The total sample size was conditioned by the economic budget available for analysing the filters in the laboratory of Microbiology at the Department of Medicine. University Jaume I (UJI), Castellón, Spain.

An intentional non-probabilistic sampling was used. Four operating rooms were chosen where the workgroups with the greatest load of laparoscopic surgery intervene, under the criterion of being the most representative of the population of patients treated in the hospital through this type of surgery. They were general and digestive surgery, emergency surgery, gynaecological surgery, and urological surgery. At random, this aforementioned order was established, with the collection of samples beginning in the general surgery operating room, continuing this sequence on successive days until the budgeted samples were completed. A nurse was in charge of collecting and processing the samples, as well as labelling the material, hiding the data from the rest of the surgical team.

Descriptive analysis summarises the characteristics of the patients and the type of surgery. Subsequently, the mean of events in the sample (sample prevalence) of the event of major interest (RT-qPCR-positive laparoscopic filters in RT-PCR-negative patients) was calculated, with a corresponding frequency exact binomial 95% confidence interval. For the inferential analysis, a Bayesian binomial estimation model was developed, aimed at estimating the prevalence of the event of interest in the reference population of the hospital. Due to the lack of data in previous studies on the prevalence of this type of event, a noninformative (1,1) beta distribution was introduced as a prior distribution. When computed with the data from the sample, the mean risk or prevalence in the reference population represented by the posterior probability of event, as well as the 95% confidence interval of posterior probabilities of the event, was obtained. Lastly, the risk of an event (positive predictive value) was calculated as an example in various specific numbers of surgical interventions to be executed. Analysis was performed with the statistical program JASP (JASP Team 2020, JASP Version 0.14.1, Amsterdam, The Netherlands) (Computer software).

## 3. Results

From April 2020 to May 2021, during the highest incidence of the pandemic outbreak, a total of 180 smoke filter systems were collected from 180 patients used in different laparoscopic surgeries. Fifty smoke filter systems were discarded due to the difficulty with handling the device and the impossibility of extracting the RNA sample. Finally, 130 filters were analysed. The study authorisation and the exemption from informed consent by the patient due to the exceptional pandemic situation were granted by the ethics and research committee of the centre (CEim-18320).

The complete sample included 59 (45%) digestive surgery patients, 49 (37%) from gynaecologic surgery, and 23 (18%) from urologic surgery. All procedures were major surgery under general anaesthesia. By sex, 62% of men and 38% of women were intervened. The mean age of the patients was 60 years (range 6–92). The age values categorised into older and younger than 60 years revealed proportions of 49% and 51%, respectively. Moreover, 47% of patients had some type of comorbidity. There were 20% urgent surgeries and 80% elective surgeries performed. There were 39% and 61% of surgeries for benign and malignant pathology, respectively. Some visceral resection was performed in 86% of the cases. There were major postoperative complications in 11% of the patients, with no postoperative mortality. The mean surgical time was 159 min from anaesthetic induction to the end of surgery, with 47% of surgeries lasting less than 120 min and 53% of surgeries lasting more than 120 min. No patient had a positive oropharyngeal test after the intervention.

In addition, no viral RNA was detected by RT-qPCR in those in-filter membranes collected from the two patients diagnosed as COVID-19-positive. On the other hand, two RT-qPCR positive in-filter membranes were found in 128 patients with SARS-CoV-2-negative results in their oropharyngeal RT-qPCR (Table 1).

The general prevalence of the risk factor was 1.5% (2/130). This estimate should be considered as not representative of the population of patients undergoing laparoscopic surgery, since the sample of patients with positive oropharyngeal RT-qPCR is underrepresented as a consequence of the restrictions imposed by the surgical safety protocol. The prevalence of negative RT-qPCR in-filter membranes for SARS-CoV-2 (negative in-filters) in COVID-19-negative patients was 97.7% (126/128). However, the result of greatest interest is represented by the prevalence of SARS-CoV-2 RT-qPCR-positive in the in-filter membranes (positive in-filters) in COVID-19-negative patients, which is shown below.

### 3.1. Analysis of the Subsample of 128 Patients with Two Positive In-Filters and Negative Oropharyngeal RT-qPCR

The prevalence of positive in-filters was 1.6% (2/128). The 95% confidence interval for the sample mean was 0.2% to 5.5%. Table 2 specifies the clinicopathological characteristics of the two cases of positive filters for SARS-CoV-2.

The Bayesian estimate is different from the frequency estimate and is based on the fact that the event analysed is present in the population, although, in the present analysis, it is unknown to what magnitude. Thus, by means of the Bayesian binomial estimation, the prevalence or posterior mean of positive filters in the population from which the analysed subsample was extracted was estimated at 2.3%, with a 95% confidence interval for larger populations with limits between 0.5% and 5.5%. From this estimation, the predictive posterior probabilities of finding *n* cases of COVID-19-negative patients with positive filters in a number (#) of surgeries are shown in Table 3.

### 3.2. RT-qPCR Analysis of In-Filter Membranes

The detection of SARS-CoV-2 in the in-filter membrane collected from the commercial smoke evacuation systems was carried out by targeting the nucleoprotein (N) N1 and N2 fragments and the envelope E fragment.

Following RNA extraction of viral content, a standard RT-qPCR procedure allowed us to detect SARS-CoV-2 RNA in two in-filter membranes collected from two patients showing negative oropharyngeal RT-qPCR (see Table 2). In the case of positive in-filter membrane 1, a positive RT-qPCR signal was observed for target gene regions N1 (Ct 37.06), N2 (Ct 38.09), and for the E target gene (Ct 35.77), with estimated viral loads of 199.4 gc/mL (genome copies per 1 mL of lysis buffer used for in-filter membrane processing), 103.7 gc/mL, and 150.2 gc/mL, respectively. In the second case, positive in-filter membrane 2, only the E target gene was amplified (Ct 35.79) with an estimated viral load of 147.8 gc/mL. Briefly, SARS-CoV-2 RNA was quantified as gc by plotting the Ct values to a standard curve built with 10-fold serial dilution of quantified plasmid controls (IDT).

For each in-filter membrane collected, we analysed the recovery efficiency of mengovirus (MgV), included as an external recovery control for RNA extraction and detection, as well as the human *RPP30* gene (IDT) for the detection of human nucleic acids, which was used as an additional RNA extraction quality control and sample integrity control.

Analysis of the recovery efficiency of MgV was investigated by spiking all in-filter membranes with a known viral concentration. The presence of MgV RNA was detected in all samples, and the recovery rate was >1% (range 3–79%) in 51% of cases. In the remaining 49%, the in-filter membrane the recovery rate was <1% (range 0.96–0.4%).

The analysis of human *RPP30* gene amplification by RT-qPCR is recommended to be used as an internal control in the case of clinical samples (oropharyngeal samples) [13] to confirm the presence of human cellular material and, therefore, to check the RNA extraction efficiency, as well as the clinical sampling process (sample integrity control). Almost all of the in-filter membranes analysed were positive for the *RPP30* gene (93%), with an average Ct of 30.7 (range 21.3–37.7). Positive RT-qPCR in-filter membranes for SARS-CoV-2 (filters 1 and 2) showed amplification for *RPP30* with Ct values of 26.25 and 33.59, respectively. For the *RPP30*-negative samples (7%), all showed a recovery efficiency of MgV > 1% (range 4–79%).

## 4. Discussion

The results of this study demonstrate that it is possible to find SARS-CoV-2 RNA traces in the peritoneal cavity in patients with a negative oropharyngeal RT-qPCR in the absence of a history of systemic infection. This could be considered an unexpected and unpredictable event. To the best of our knowledge, only one study [8] reported similar findings. Bogani et al. [8] reported positive RT-qPCR amplification for SARS-CoV-2 in a swab test performed in the endotracheal tube and trocar valve filter of one patient from a small sample of 17 gynaecological interventions. The prognostic simulation shown in Table 3 allows us to confirm that this finding is not excessively frequent, but it is not exceptional either. For example, the probability of finding positive RT-qPCR laparoscopic filters for SARS-CoV-2 when 10 laparoscopies are performed in patients with negative oropharyngeal RT-qPCR is 20%, according to the prevalence of positive RT-qPCR in-filter membranes found in our study. This probability increases with the number of surgeries performed and would also increase in populations with a higher SARS-CoV-2 prevalence.

During the pandemic, there have been numerous surgical societies of different specialties that, after a meeting of experts, have recommended a series of measures for the protection aimed at both patients and surgical teams (PPE, laminar ventilation, PCR for all surgical patients, and intervention in positive patients only in urgent situations) [9,10,15]. However, these recommendations, although logical, did not have any scientific support because there was no proven evidence of the behaviour of this new disease.

Some authors have recently found the presence of SARS-CoV-2 RNA in the faeces of infected patients [5]. This finding has also been described in peritoneal dialysis samples and in patients treated in urgent surgeries during the period of pneumonia and fever [4]. However, in other patients that were also treated during the period of viral infection, the presence of the virus in the peritoneal cavity has not been demonstrated [16]. It is evident that we know very little about the evolution of the disease once the patient is infected.

Although our results imply that the virus could be found in the abdominal cavity of patients in whom the preoperative oropharyngeal RT-qPCR was negative, they do not clarify the path that the pathogen followed until contaminating the surgical field. It is also not possible to determine whether the oropharyngeal RT-qPCRs were false negatives, or whether the RT-qPCR failed to identify patients with asymptomatic COVID at the time of surgery. It is possible to propose several hypotheses: SARS-CoV-2 was present in the natural fluid of the peritoneum; viral contamination came from blood or fluids released during the surgical act; SARS-CoV-2 was lodged in the organs that were sectioned or resected; a combination of the above. Therefore, this prevalence study cannot clarify either the causes or the pathogenesis of this finding.

In the present study, we detected one SARS-CoV-2 positive (in-filter membrane 1) with RT-qPCR amplification for all three targets (N1, N2, and E) tested and a presumptive positive one (in-filter membrane 2), in which only target gene E was amplified. To corroborate our results, we included two analytical process controls. On the one hand, the sampling integrity was controlled for, using the human *RPP30* gene, which is normally used when running oropharyngeal RT-qPCR tests. We observed *RPP30* gene amplification in 93% of the in-filter membranes analysed, meaning that sufficient human cellular material was extracted from each in-filter membrane. For those in-filter membranes lacking *RPP30* amplification (7%), several interpretations could be considered, such as a loss of RNA and/or RNA degradation as a result of the improper extraction of nucleic acid from the in-filter membranes, the absence of sufficient human cellular material due to poor collection or loss of specimen, and reagent or equipment malfunction. Nevertheless, failure to detect the *RPP30* gene is possible and does not preclude the potential amplification of SARS-CoV-2 genes [16]. In any case, due to the complexity of our sample nature (in-filter membrane), a second process control, MgV, was included in our study. We were able to detect MgV RNA in all samples with a recovery efficiency of MgV > 1% in 51% of the cases, which is interpreted as an acceptable value according to previous work [15]. However, in 49% of the in-filter membranes, the recovery rate was <1%, which could be interpreted because of RNA loss and/or RNA degradation during in-filter handling or RNA extraction processes. Nevertheless, it is important to point out that, for all these samples with lower MgV recovery (<1%), *RPP30* amplification was detected. 

In our study, only two patients with positive oropharyngeal RT-qPCR and negative laparoscopic in-filter RT-qPCR membranes were operated on from the emergency department. This result should be considered anecdotal due to the low number of sick patients operated on due to the restrictions imposed by the surgical safety protocol of our hospital. A high number of RT-qPCRs should be performed on laparoscopic in-filter membranes of infected patients at different stages of the disease to be able to assess when the virus reaches the abdominal cavity. However, a study of these characteristics is difficult to carry out in circumstances of high transmissibility in the population due to the hypothetical risk of contagion from operating room personnel, as well as the probable negative consequences for the patient when operating in the phase of active disease [17].

In any case, given that the risk of finding SARS-CoV-2 infective particles in the abdominal cavity of patients with negative nasopharyngeal RT-qPCR exists, and the fact that it seems to be relatively high in laparoscopic surgery, additional safety measures should be taken for healthcare personnel in the operating room that would not have been planned previously. In the first place, the entry and exit of manipulation and optical forceps through the abdominal trocars should be minimised in order to avoid pneumoperitoneum leaks; it would also possibly be necessary to monitor the existence of SARS-CoV-2 in the form of aerosols in the operating room. The number of professionals inside the operating rooms should also be limited, and they should use Fpp2 protection masks on a mandatory basis.

On the other hand, it must be taken into account that the possible evacuation of aerosols contaminated by particles of SARS-CoV-2 during laparoscopy is done in a much more controlled way than in a laparotomy; therefore, the initial recommendation to try to minimise the number of laparoscopic surgeries procedures during the pandemic might not be correct, since a laparoscopy with controlled evacuation of the pneumoperitoneum surely offers greater safety for both the patient and the surgeon than a laparotomy.

The main weaknesses of the study included the inherent biases of the single-centre nature of our investigation. However, the main strength of our investigation remains in the cross-sectional prospective study design and the different types of surgeries and surgeons evaluated during the different waves of pandemic COVID-19 outbreak.

In conclusion, the present study provides evidence suggesting that the airborne transmission of SARS-CoV-2 particles from smoke evacuation of aerosols carrying viral particles during laparoscopy should be strongly considered. Oropharyngeal RT-qPCR of patients can fail to identify asymptomatic COVID-19 patients and, therefore, health staff must take precautions during surgical interventions. The risk of transmission appears to increase as the number of surgeries performed increases.

## Figures and Tables

**Table 1:** Two-by-two table between the risk factor (oropharyngeal RT-qPCR) and peritoneal disease (RT-qPCR in-filters membranes).

Peritoneal COVID (In-Filter RT-qPCR)	COVID-19 Risk Factor (Oropharyngeal RT-qPCR)		Total
	Positive	Negative	
**Positive**	0	2	2
**Negative**	2	126	128
**Total**	2	128	130

**Table 2:** Clinicopathological characteristics of the two positive laparoscopic filters.

	Positive In-Filter Membrane 1	Positive In-Filter Membrane 2
**Amplified RT-qPCR target genes**	N1, N2, and E	E
**Date**	April 2020	May 2020
**Age**	60	42
**Sex**	Men	Woman
**Urgent surgery**	No	No
**Type of surgery**	Gastrectomy	Partial nephrectomy
**Visceral resection**	Yes	Yes
**Comorbidities**	Yes	No
**Complications**	No	No
**Duration of surgery**	>120 min	>120 min
**Final diagnostic**	Gastric adenocarcinoma	Renal Carcinoma

**Table 3:** Posterior predictive probabilities of positive filters corresponding to a distribution Beta-Binomial with parameters (#, 2, 128).

*n* Cases with Filters +	*P* (%) in # 100 Surgeries	*P* (%) in # 50 Surgeries	*P* (%) in # 25 Surgeries	*P* (%) in # 10 Surgeries
0	18	37	59	80
>0	82	63	41	20
1	24	32	29	17
2	21	18	9	2
3	15	8	2	<1
4	10	3	<1	<1
5	6	1	<1	<1
6	3	<1	<1	<1
7	2	<1	<1	<1
8	1	<1	<1	<1

*P*: posterior predictive probabilities in # patients or surgeries.

## Data Availability

The data that support the findings of this study are available from the corresponding author upon reasonable request.
